# Changing Pattern of Human Listeriosis, England and Wales, 2001–2004

**DOI:** 10.3201/eid1209.051657

**Published:** 2006-09

**Authors:** Iain A. Gillespie, Jim McLauchlin, Kathie A. Grant, Christine L. Little, Vina Mithani, Celia Penman, Christopher Lane, Martyn Regan

**Affiliations:** * Health Protection Agency, London, United Kingdom;; † Health Protection Agency North West, Liverpool, United Kingdom

**Keywords:** *Listeria monocytogenes*, surveillance, outbreaks, epidemiology, research

## Abstract

Microbiologic and epidemiologic data on 1,933 cases of human listeriosis reported in England and Wales from 1990 to 2004 were reviewed. A substantial increase in incidence occurred from 2001 to 2004. Ten clusters (60 cases), likely to represent common-source outbreaks, were detected. However, these clusters did not account for the upsurge in incidence, which occurred sporadically, predominantly in patients >60 years of age with bacteremia and which was independent of sex; regional, seasonal, ethnic, or socioeconomic differences; underlying conditions; or *Listeria monocytogenes* subtype. The reasons for the increase are not known, but since multiple *L. monocytogenes* strains were responsible, this upsurge is unlikely to be due to a common-source outbreak. In the absence of risk factors for listeriosis in this emerging at-risk sector of the population, dietary advice on avoiding high-risk foods should be provided routinely to the elderly and immunocompromised, not just to pregnant women.

## Abstract 2

Disease has reemerged, mainly in patients ≥60 years of age with bacteremia.

The bacterium *Listeria monocytogenes* and the disease listeriosis were first recognized in laboratory animals in 1924 (*1*). The disease also affects humans, most commonly the unborn, neonates, the immunocompromised, and the elderly. Listeriosis manifests primarily as abortion, septicemia, or central nervous system (CNS) infections, with a high case-fatality rate in all patient groups. Although most cases are foodborne, the epidemiology is complex (*2*). The ubiquitous nature of the bacterium, together with a varied incubation period (1 to >90 days), means that identifying specific food vehicles can be problematic (*3*).

Human listeriosis was very rare in England and Wales during the 1960s and 1970s but increased at the end of the 1980s. From 1987 to 1989, the incidence doubled, probably due to the consumption of contaminated pâté. After a specific brand of pâté was withdrawn from retail sale and warnings were issued to pregnant women and the immunocompromised to avoid eating this food, the incidence declined (*4*). Throughout the 1990s, the average annual total of cases was 110, but the numbers increased to 146, 136, 237, and 213 cases in 2001, 2002, 2003, and 2004 respectively (*5*). We describe preliminary surveillance data on human listeriosis in England and Wales from 1990 to 2004 and speculate on reasons for the upsurge.

## Materials and Methods

The Health Protection Agency Centre for Infections (CFI) coordinates routine surveillance of human listeriosis in England and Wales. Case ascertainment is by the voluntary reporting of laboratory-diagnosed cases from microbiology laboratories through an electronic reporting system, or by referral of cultures for identification and subtyping. Epidemiologic and microbiologic data from both systems are combined, checked for duplication, and stored in a database. Additional demographic and clinical data are sought from the responsible consultant medical microbiologists and local health protection teams with a standard questionnaire.

*L. monocytogenes* isolates from patients with clinical cases, food, and the environment referred to CFI are confirmed phenotypically (*6*) or by PCR (*7*). Isolates are characterized by serotyping (*8**,**9*), phage typing until 2003 (*10*), amplified fragment-length polymorphism (AFLP) analysis since 2002 (*11*), and pulsed-field gel electrophoresis since 2003 on selected isolates (*12*).

For surveillance purposes, a patient with listeriosis was defined as one with a compatible illness from whom *L. monocytogenes* was isolated from a normally sterile site, usually blood or cerebrospinal fluid (CSF). Cases were categorized as pregnancy associated (all maternal-fetal patients and neonatal patients; a mother-baby pair was considered 1 case-patient) and nonpregnancy associated (in a patient >1 month of age). Case-patients were categorized further into those with CNS infections (*L. monocytogenes* isolated from CSF or brain tissue, clinical evidence of infection of this organ, or both); bacteremia in the absence of CNS infections (*L. monocytogenes* isolated from blood but not from CNS and without clinical evidence of CNS infection); and other conditions not included in the previous 2 categories.

Data manipulation was undertaken in Microsoft Access 2003 (Microsoft Corporation, Redmond, WA, USA) and MapInfo version 8.0 (MapInfo Corporation, Troy, NY, USA). Ethnicity (categorized as ethnic or nonethnic) was assigned by using patients' names (surname and first name when available); patients' ages were grouped into 10-year bands. Indexes of deprivation for England (2004 [13]) and Wales (2005 [14]), ranked and arranged into quintiles (1 = most deprived and 5 = least deprived areas) and linked to patients postal codes, were used as an approximate marker for patients' socioeconomic status. Patients' postal codes were also used as a marker for patients' residency. Internet searches were used to determine if residential care homes were situated in that postal code area or whether the housing was purely residential.

Data analysis was performed with Microsoft Excel, EpiInfo version 6.04b (Centers for Disease Control and Prevention, Atlanta, GA, USA) and Stata version 8.2 (StataCorp, College Station, TX, USA). Age-specific denominator data from 1990 to 2004 were obtained from the Office for National Statistics. Relative proportions and changes in relative proportions with time were compared by using the χ^2^ test and the χ^2^ test for trend, respectively. Point estimates of relative risks (RRs) with accompanying 95% confidence intervals (CIs) and significance tests were also calculated.

## Results

### All Reported Cases

From January 1, 1990, to December 31, 2004, a total of 1,933 reported cases of human listeriosis in England and Wales fulfilled the case definition. Of these 1,377 (71%) were reported through the electronic surveillance system, 1,592 (82%) by isolate referral and 1,068 (55%) by both means. During the study period, the proportion of isolates referred did not change (χ^2^ for trend p = 0.94); the proportion of electronic reports received increased slightly (χ^2^ for trend p = 0.04). A total of 1,776 patients were admitted to the hospital. For 1,187 patients for whom outcome data were available, 522 (44%) died. From 1990 to 2000, the mean annual incidence was 2.13 cases/million/year (95% CI 2.01–2.25), which increased significantly to 3.47 cases/million/year (95% CI 3.22–3.73) from 2001 to 2004 (RR 1.39, 95% CI 1.31–1.47, p<0.001).

### Clustered Cases

Epidemiologic and subtyping analysis identified 10 clusters of cases, which affected 60 patients and likely reflected common-source outbreaks (*15**,**16*); these are summarized in Table 1. When these cases were excluded, a significant increase in disease in 2001–2004 compared with 1990–2000 remained (RR 1.34, 95% CI 1.26–1.42, p<0.001). Subsequent analysis is confined to 1,873 sporadic cases unless otherwise indicated.

**Table 1:** Clusters of human listeriosis, England and Wales, 1990–2004*

Year	Area	No. cases	Pregnancy associated	*Listeria monocytogenes* type				Vehicles of infection
				Serovar	AFLP	Phage	PFGE	
Clusters probably or likely to be common-source foodborne outbreaks								
1999a	NE England	4	0	4b	ND	ND	ND	Hospital sandwiches
2003	NE England	17	11	4b	V	A	2	Butter
2003	NE England	18	0	4b	I	G	1	None identified
2003	S Wales	2	0	1/2a	XI	Y	L	Hospital sandwiches
2003b	SW England	5	5	1/2a	III	Y	A	Hospital sandwiches
2004	E Midlands	6	0	4b	I	ND	E	None identified
				4b	IV	ND	M	None identified
				4b	V	ND	J	None identified
2004	SE England	2	0	4b	I	ND	A	Hospital sandwiches
				4b	V	ND	B	Not identified
Episodes of neonatal cross-infection								
1990	SE England	2	2	4b	ND	ND	ND	Contact between patients within a delivery suite
1997	SE England	2	2	4b	ND	H	ND	Contact between patients within a delivery suite
1998	SE England	2	2	1/2a	ND	I	ND	Contact between patients within a delivery suite

*AFLP, amplified fragment-length polymorphism; PFGE, pulsed-field gel electrophoresis; NE, northeast; ND, not done; NT, nontypable; SW, southwest; SE, southeast. Two clusters have previously been described: a (*15*) and b (*16*).

### Trends in Sporadic Cases

**Figure 1:**
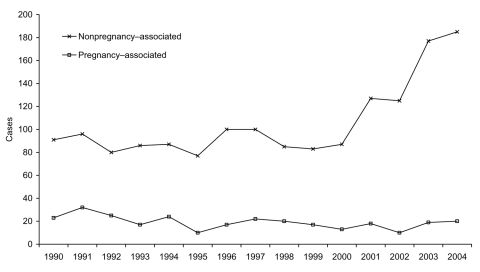
Sporadic cases of listeriosis reported in England and Wales, 1990–2004.

In 1990, sporadic nonpregnancy-associated listeriosis accounted for 80% of the 114 cases reported, and in 2004 for 90% of the 205 cases reported (χ^2^ for trend p<0.001; Figure 1). A total of 510 (44%) of 1,155 of the nonpregnancy-associated patients and 29 (10%) of 287 of the sporadic pregnancy-associated patients died.

**Figure 2:**
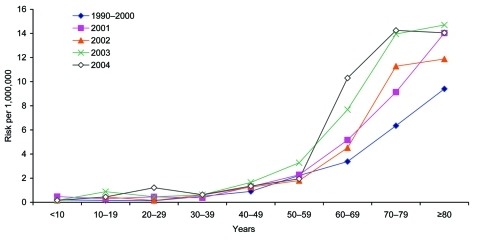
Risk for sporadic nonpregnancy-associated listeriosis by age group, England and Wales, 1993–2004. Individual data shown for years 2001–2004.

Data on patients' age were available for 1,543 (97%) of the 1,586 nonpregnancy-associated cases. In 2001–2004, the risk for nonpregnancy-associated listeriosis in persons >60 years of age increased by almost half (RR 1.49, 95% CI 1.39–1.60, p<0.001) compared with 1990–2000 (Figure 2). After 2000, the risk among 70- to 79-year-olds (RR 1.32, 95% CI 1.20–1.45, p<0.001) and >80-year-olds (1.51, 95% CI 1.33–1.71, p<0.001) was significantly higher than for 60- to 69-year-old patients. Sporadic patients >60 years of age were more likely to die (405 [49%] of 828) than those <60 years of age (97 [31%] of 315, χ^2^ p<0.001), and the likelihood of death increased with increasing age in this group (χ^2^ for trend, p = 0.01).

**Figure 3:**
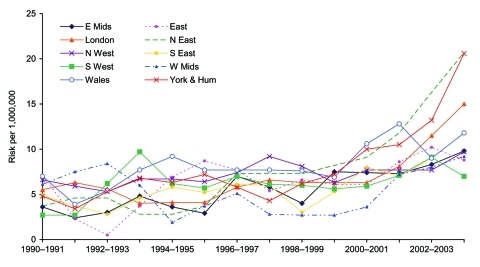
Biannual risk for sporadic nonpregnancy-associated listeriosis in patients >60 years of age, by region, England and Wales, 1990–2004. E Mids, East Midlands; East, East of England; N East, Northeast England; N West, Northwest England; S East, Southeast England; S West, Southwest England; W Mids, West Midlands, Wales; York & Hum, Yorkshire and the Humber.

Data on sex were available for 1,542 (99%) of the 1,543 nonpregnancy-associated patients for whom age was also available. The increased risk in persons >60 years of age during 2001–2004 compared with the risk in 1990–2000 was observed in both men (RR 1.47, 95% CI 1.34–1.62, p<0.001) and women (RR 1.49, 95% CI 1.34–1.66, p<0.001) and occurred in most regions of England and in Wales (Figure 3).

Specimen collection dates were available for 1,088 (99%) of 1,102 nonpregnancy-associated patients >60 years of age; 503 (46%) cases occurred from July to October. This pattern did not differ for nonpregnancy-associated patients >60 years of age reported from 2001 to 2004 (206 [47%] of 442) compared with those reported from 1990 to 2000 (297 [46%] of 646, χ^2^ p = 0.84).

Serotyping data were available for 889 (81%) of the 1,102 nonpregnancy-associated patients >60 years of age. Serotypes 4b (436 [49%]) and 1/2 (430 [48%]) accounted for most cases from 1990 to 2004; this proportion did not change significantly during the study period (χ^2^ for trend, p = 0.13). No significant differences were observed between patients infected with serotypes 1/2 or 4b in terms of age distribution or death. AFLP typing, applied to cultures collected from 2002 to 2004 and used in conjunction with serotyping, was available for 267 (99%) of 269 nonpregnancy-associated patients >60 years of age. Fourteen, 18, and 16 different subtypes were reported in 2002, 2003, and 2004 respectively; 2, 4, and 4 subtypes, respectively, were unique to these years. Ten subtypes were responsible for 241 (90%) of cases, and no individual type occurred more frequently than 22% in any individual year or among all cases.

Data on underlying illness were available for 830 (75%) of 1,102 nonpregnancy-associated patients >60 years of age; this proportion did not differ during 1990–2000 (90%) and 2001–2004 (89%). A single underlying condition was reported for 635 (77%) patients, >1 (12%) underlying condition was reported for 97 patients, the specific underlying condition was not recorded for 10 patients (1%), and 88 patients had no underlying condition. No significant change in the underlying conditions reported for patients occurred in 1990–2000 compared with 2001–2004 (Table 2).

**Table 2:** Underlying conditions reported for sporadic nonpregnancy-associated listeriosis patients >60 years of age, England and Wales, 1993–2004

Classification	1993–2000, n (%)	2001–2004, n (%)	Total
Cancers	173 (42)	143 (43)	316
Autoimmune disorders	53 (13)	46 (14)	99
Cardiovascular disorders	54 (13)	39 (12)	93
Alcohol-related disorders	14 (3)	12 (4)	26
Renal disorders	12 (3)	16 (5)	28
Diabetes	11 (3)	10 (3)	21
Hepatic and biliary disorders	9 (2)	4 (1)	13
Immunosuppressed	1 (0)	4 (1)	5
Postoperative	2 (0)	3 (1)	5
Multiple pathologic conditions	59 (14)	38 (12)	97
Other pathologic conditions	19 (5)	10 (3)	29
Not specified	6 (1)	4 (1)	10
Total	413	329	742

**Figure 4:**
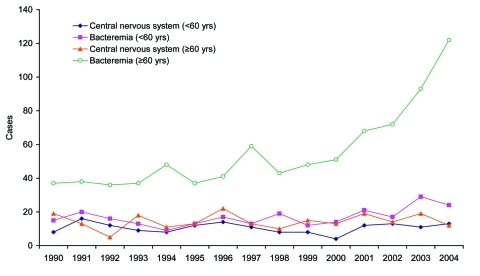
Sporadic nonpregnancy-associated listeriosis in patients with central nervous system infections and bacteremia alone, England and Wales, 1990–2004.

Among all nonpregnancy-associated patients, 380 (24%) had evidence of CNS infections, 1,114 (70%) had bacteremia without CNS infections, 59 (4%) had other conditions, and 33 (2%) could not be categorized. The proportion of nonpregnancy-associated patients >60 years of age with bacteremia alone increased significantly from 2001–2004 compared with 1990–2000 (85% vs. 76%, χ^2^ p = 0.0004) (Figure 4). This difference in proportion was not observed in nonpregnancy-associated patients <60 years of age (65% vs. 59%, χ^2^ p = 0.3). Among nonpregnancy-associated patients >60 years of age with CNS involvement, the proportion with an accompanying blood-culture isolate from 2001 to 2004 (44 [73%] of 60) was not significantly different than the rate from 1990 to 2000 (98 [65%] of 151, χ^2^ p = 0.24). Among all 264 pregnancy-associated patients, the proportion with a blood-culture isolate from 2001 to 2004 (40 [63%] of 64) was not significantly different from that in 1990–2000 (101 [51%] of 200, χ^2^ p = 0.09).

Patients' names were available for 1,092 (99%) of 1,102 nonpregnancy-associated patients >60 years of age; these were used as a marker for ethnicity. Most patients (1,040, 95%) were classified as nonethnic on the basis of their name. This proportion did not change from 1990–2000 (623 [96%] of 650) to 2001–2004 (417 [94%] of 442, χ^2^ p = 0.44).

Patients' postal codes were available for 634 (58%) of 1,102 nonpregnancy-associated patients >60 years, and indexes of deprivation were determined for 563 (89%). The proportion of patients who fell into the quintiles of deprivation 1–5 did not change during the surveillance period (χ^2^ for trend p = 0.57, 0.69, 0.64, 0.05, and 0.14, respectively).

Internet searches of the areas covered by the postal codes of the 634 nonpregnancy-associated patients >60 years of age showed that, when genuine postal codes were supplied (628, 99%), most (580, 92%) did not contain a residential care home. This proportion did not differ from 2001–2004 (27 [9%] of 309) when compared with 1990–2000 (21 [7%] of 319, χ^2^ p = 0.31).

## Discussion

Routine surveillance of human listeriosis in England and Wales showed an upsurge in cases such that the annual incidence is now comparable with other European countries with higher incidence (*17*). The clinical manifestations have also changed: bacteremia in older patients without CNS involvement predominates. Several confounding factors could explain the increase in cases and changes in signs and symptoms.

Changes in reporting or referral could have accounted for the observed increase in incidence. The surveillance of listeriosis in England and Wales is passive, and such systems are prone to both underascertainment and pseudo-outbreaks following increased interest in the public health community. Although reporting artifacts cannot be excluded, we are unaware of increased interest in listeriosis from 2001 onwards. Furthermore, reporting and referrals did not change enough to explain the increase.

Improvements in laboratory methods (especially in the isolation of *L. monocytogenes* from blood) or changes in local clinical practice (e.g., more detailed investigations of patients with acute febrile illness seeking primary care) might explain the increase in cases diagnosed or the altered clinical manifestations. We are unaware of substantial changes in blood culture techniques used in England and Wales in the past decade that would increase the diagnosis of listeriosis. Furthermore, although the introduction of mandatory reporting of methicillin-resistant *Staphylococcus aureus* bacteremia in England in 2001 has led to an increase in blood cultures being taken, this is insufficient to explain the increase or shift in clinical manifestations described here (*18**,**19*). Further evidence that the increase was not due to improved diagnostics is the absence of statistically significant increase in the isolation of *L. monocytogenes* from blood cultures from patients with CNS infections or from pregnancy-associated patients.

Demographic changes in the population might have resulted in an overrepresentation of patients from particular age groups without a true increase in risk. Life expectancy in the United Kingdom is increasing; therefore, an increase in listeriosis in older patients is likely to occur. However, calculations controlling for the changing age structure in England and Wales during the surveillance period generates a consistent increase in risk among those >60 years of age. Medical advances have resulted in the UK population's surviving for longer with chronic conditions (*20*) with a likely increased susceptibility to listeriosis. While the denominator data required to examine such changes in detail are unavailable, changes would be unlikely to result in an almost 3-fold increase in a single patient age group in a short period without a concomitant increase in younger patients with similar underlying conditions.

Changes in the pathogenicity of *L. monocytogenes* might explain the change in disease manifestations. However, the increase has been due to multiple subtypes, which makes this unlikely. Furthermore, since the upsurge was confined to a restricted patient age group, it is more likely to reflect increased incidence through higher exposure that accompanies behavioral changes.

Having examined the most plausible sources of bias, we believe that the observed upsurge and altered clinical manifestations are genuine. Indeed, historical data suggest that the current picture merely represents a continued shift in the epidemiology and clinical manifestations of *L. monocytogenes* infection in England and Wales (Table 3) (*4**,**21*).

**Table 3:** Risk perspective for listeriosis in England and Wales, 1967–2004

Years	Cases per year	Percentage		Outbreaks	Reference
		Pregnancy associated	<60 y of age (bacteremia)		
1967–1985	<75–136	33	31 (16)	Some clusters (foodborne?)	(*4*)
1987–1989	237–278	40	–	50% in 1 outbreak (pâté)	(*21*)
1990–2000	87–128	19	67 (49)	Limited clusters	This study
2001–2004	136–237	11	73 (58)	Largely sporadic, some clusters	This study

The routine epidemiologic and microbiologic data collected for cases of listeriosis in England and Wales are not exhaustive; therefore, our retrospective examination of the factors that have contributed to this upsurge is preliminary. Nevertheless, we have demonstrated that the upsurge is independent of sex; regional, seasonal, ethnic, or socioeconomic differences; underlying conditions; or *L. monocytogenes* subtypes. Furthermore, most older patients in the surveillance period did not reside in care homes and were therefore unlikely to have changed exposure to institutional catering in such settings. UK food consumption/expenditure data also suggest that no major shift in the consumption of major food groups by the older population has occurred in recent years to explain the increase (*22*).

Investigations are continuing to establish the causes of the increase and include application of discriminatory subtyping of *L. monocytogenes* isolates, coupled with the collection of standardized clinical and epidemiologic data for all patients. Hopefully, such steps will facilitate outbreak detection and help identify their cause, as well as enable investigations of factors specific to *L. monocytogenes* subtypes among sporadic cases. However, analytical epidemiology (including case-control studies) and molecular fingerprinting of isolates have not always successfully identified the appropriate interventions to control outbreak-associated and sporadic listeriosis, which suggests that new approaches to investigation are required. Therefore, in the absence of risk factors for listeriosis in this emerging at-risk sector of the population, dietary advice on the avoidance of high-risk foods should be provided routinely to the elderly and immunocompromised and not just pregnant women.
